# Metabolite coupling analysis and metabolite-flux coupling analysis of genome-scale metabolic models

**DOI:** 10.3389/fbinf.2026.1859473

**Published:** 2026-07-01

**Authors:** Mingyuan Tian

**Affiliations:** 1 Department of Chemical and Biological Engineering, University of Wisconsin-Madison, Madison, WI, United States; 2 Independent Researcher, Albuquerque, NM, United States

**Keywords:** coupling analysis, metabolomics, modules, omics data analysis, proteomics, transcriptomics, multi-omics

## Abstract

**Background:**

Genome-scale metabolic models (GEMs) provide detailed representations of metabolic networks. Flux Coupling Analysis (FCA) is widely used for analyzing dependencies between reaction fluxes in GEMs.

**Results:**

We introduce Metabolite Coupling Analysis (MCA) and Metabolite-flux Coupling Analysis (MetFCA), two methods that extend FCA concepts from reactions to metabolites and metabolite-reaction pairs, enabling the identification of condition-specific modules for omics (e.g., transcriptomics, proteomics, and metabolomics) data analysis.

**Conclusion:**

MCA and MetFCA, together with FCA, provide a unified framework for generating condition-specific modules in GEMs. These modules exhibit clearer biological functions than those generated by statistical, data-driven approaches. A case study demonstrates the use of gene modules to analyze transcriptomics data in the influenza-infected Calu-3 cell line.

## Introduction

Genome-scale metabolic models (GEMs) provide a rich source of information on reaction stoichiometry and metabolite connectivity in organisms. In the past 20 years, many GEMs have been reconstructed for various organisms, including *E*. *coli* (Reed et al., 2003; Orth et al., 2011), *S*. *cerevisiae* (Mo et al., 2009), *B*. *subtilis* (Oh et al., 2007), and humans (Duarte et al., 2007; Thiele et al., 2013; Blais et al., 2017). GEMs have many applications including predicting flux distributions under various conditions (Segrè et al., 2002; Orth et al., 2010; Kim and Reed, 2012; Tian and Reed, 2018) and identifying strain design strategies to produce chemicals of interest (Burgard et al., 2003; Pharkya and Maranas, 2006; Kim and Reed, 2010; Kim et al., 2011; Cotten and Reed, 2013). Therefore, it is important to learn about the properties of GEMs.

Flux Balance Analysis (FBA) (Orth et al., 2010) is the foundational computational method used to study GEMs by calculating growth rates and flux distributions. Flux is the rate at which a specific reaction occurs, usually expressed in *mmol/gDW/h* (millimoles of metabolite produced or consumed per gram of cellular dry weight per hour). Metabolic networks are mathematically represented by a stoichiometric matrix (*S*). In a system with *m* compounds and *n* reactions, each of the *m* rows represents a unique compound, and each of the *n* columns represents a specific reaction. The entries within the matrix correspond to the stoichiometric coefficients of the metabolites participating in each reaction. By applying various constraints and solving this mathematical problem, FBA determines the flux distributions.

To understand the relationships between these fluxes, researchers use Flux Coupling Analysis (FCA) (Burgard et al., 2004). For every pair of reactions, FCA determines whether two reactions are (1) fully coupled (2) partially coupled (3) directionally coupled or (4) uncoupled. However, as the name suggests, FCA is performed only on reaction fluxes, not on metabolites. Metabolite Concentration Coupling Analysis (MCCA) (Nikolaev et al., 2005) is a method to study relationships between metabolite concentrations in GEMs. However, the focus of MCCA is on metabolite concentration, rather than the flux through the metabolite and hence it cannot link metabolite activity to flux distributions.

In this paper, we extend FCA from a reaction perspective to a metabolite perspective. We develop Metabolite Coupling Analysis (MCA) and Metabolite-flux Coupling Analysis (MetFCA) to evaluate the dependencies between each pair of metabolites and each pair of metabolites and reactions in GEMs, respectively. These methods build upon earlier foundational work (Tian, 2018). Recently, Seyis et al. proposed the Flux-sum Coupling Analysis (FSCA) (Seyis et al., 2025) which utilizes the same mathematical formulation as that of MCA. In the Results section, we provide a comparative analysis of these approaches using the same GEM.

A primary application of these methods is the generation of functional modules. In the context of this paper, a module is strictly defined as a cluster of fully coupled entities, explicitly excluding any directional or partial coupled or uncoupled pairs. The composition of these modules depends on the specific method applied: FCA identifies reaction modules, MCA identifies metabolite modules, and MetFCA identifies metabolite-reaction modules. Furthermore, if gene-protein-reaction (GPR) associations are available for the GEMs, both FCA and MetFCA can be extended to yield gene and metabolite-gene modules, respectively. Ultimately, the combined application of these three coupling analysis provides a robust framework for analyzing omics datasets.

These mathematically derived modules represent a functional alternative to widely used knowledge-based approaches (Khatri et al., 2012; Nguyen et al., 2024), which rely on static pathway annotations from repositories like KEGG (Kanehisa, 2000) and MetaCyc (Karp, 2002). Rather than relying on these fixed lists of curated gene and metabolite sets, our method defines modules dynamically based on flux distributions under various constraints, such as specific experimental conditions or gene knockouts (see *Methods* section). Notably, during the module generation process, FCA, MCA and MetFCA deliberately exclude blocked reactions (inactive reactions that always carry zero flux). By grouping only functionally active genes and metabolites together, our method provides researchers with a novel way to uncover condition-specific metabolic functionality that static annotations inherently miss. Methodological differences between traditional knowledge-based approaches and the FCA/MCA/MetFCA approach for omics data analysis are shown in Table 1.

**TABLE 1:** Methodological differences between traditional knowledge-based approaches and the FCA/MCA/MetFCA approach for omics data analysis.

Characteristic	Traditional knowledge-based approaches	Proposed approach (FCA/MCA/ MetFCA)
Module definition	Static, predefined sets from repositories (e.g., KEGG, MetaCyc)	Dynamic, condition-specific modules derived from metabolic flux relationships
Reaction filtering	Often retain genes and metabolites associated with blocked (inactive) reactions	Deliberately filters out blocked reactions
Primary output	Enriched pathways	Enriched functionally active modules

## Methods

### Flux coupling analysis (FCA)

FCA (Burgard et al., 2004) maximizes and minimizes the ratio of fluxes through two reactions l and k (Equation 1a), as shown below.

$$\max/\min\;\frac{v_{l}^{+}}{v_{k}^{+}}\text{ or }\left(\frac{v_{l}^{-}}{v_{k}^{+}}\right)\text{ or }\left(\frac{v_{l}^{+}}{v_{k}^{-}}\right)\text{ or }\left(\frac{v_{l}^{-}}{v_{k}^{-}}\right)\;\forall k,l\in \text{Reaction}$$ (1a)

s.t.

$$\sum_{j\in \text{Reaction}}S_{\text{ij}}\cdot \left(v_{j}^{+}-v_{j}^{-}\right)=0\;\forall i\in \text{Metabolite}$$ (1b)

$$0\leq v_{j}^{+}\leq UB_{j}\;\forall j\in \text{Reaction}$$ (1c)

$$0\leq v_{j}^{-}\leq -LB_{j}\;\forall j\in \text{Reaction}$$ (1d)

Equation 1b is the steady-state mass balance constraint, meaning there is no accumulation of each metabolite i in the cell. $S_{ij}$ is the stoichiometric coefficient for metabolite i and reaction j, and $v_{j}$ is the flux through reaction j. Every reaction $v_{j}$ is split into a forward reaction $v_{j}^{+}$ and a backward reaction $v_{j}^{-}$ . Therefore, $v_{j}$ is equal to $v_{j}^{+}-v_{j}^{-}$ . Equations 1c,d, [Disp-formula e1d] are the enzyme capacity constraints. For each reaction j, $v_{j}$ is between upper bound ( $UB_{j}$ ) and lower bound ( $LB_{j}$ ), that is, $LB_{j}\leq v_{j}\leq UB_{j}$ . Since each reaction $v_{j}$ is split into a forward reaction and a backward reaction, $LB_{j}\leq v_{j}\leq UB_{j}$ is decomposed into Equations 1c,d, [Disp-formula e1d]. For reversible reactions, the upper bounds ( $UB_{j}$ ) and lower bounds ( $LB_{j}$ ) are set to 100,000 *mmol/gDW/h* and −100,000 *mmol/gDW/h*, respectively. For irreversible reactions, the upper bounds ( $UB_{j}$ ) and lower bounds ( $LB_{j}$ ) are set to 100,000 *mmol/gDW/h* and 0 *mmol/gDW/h*, respectively. For the exchange reactions of substrates, $LB_{j}$ is set to −100,000 *mmol/gDW/h*. For example, if a glucose aerobic medium is used, the lower bound for the exchange flux for glucose, oxygen, and other minimal medium components (e.g., water, hydrogen, and nitrogen) is set to −100,000 *mmol/gDW/h*. The flux for ATP maintenance is set to 0 mmol/gDW/h. The objective function for FCA is a non-linear flux ratio, which is challenging to solve. Details on how to transform this non-linear formulation to a linear programming problem is shown in Burgard et al. (2004). The problem was solved using the CPLEX solver in GAMS.

### Metabolite coupling analysis (MCA)

We define the flux-sum as the absolute value of the sum of fluxes through one metabolite, including all incoming and outgoing fluxes, weighted by the stoichiometric coefficients. This is slightly different from the definition by Kim et al. (2007). The mathematical definition of the flux-sum through metabolite k used in this paper is $m_{k}=\sum_{j\in Reaction}\left|S_{kj}v_{j}\right|$ , where $S_{kj}$ is the stoichiometric coefficient for metabolite k and reaction j, and $v_{j}$ is the flux through reaction j. If every reaction is split into a forward reaction and a backward reaction, that is, $v_{j}=v_{j}^{+}-v_{j}^{-}$ , then flux-sum can also be written as $m_{k}=\sum_{j\in Reaction}\left|S_{kj}\left(v_{j}^{+}-v_{j}^{-}\right)\right|$ . MCA, following the same rationale of FCA, evaluates the ratio of the flux-sums for two metabolites l and k (Equation 2a). MCA is formulated as the following optimization problem:

$$\max/\min\frac{m_{l}}{m_{k}}\;\forall l,k\in \text{Metabolite}$$ (2a)

s.t.

$$\sum_{j\in \text{Reaction}}S_{\text{ij}}\cdot \left(v_{j}^{+}-v_{j}^{-}\right)=0\;\forall i\in \text{Metabolite}$$ (2b)

$$0\leq v_{j}^{+}\leq UB_{j}\;\forall j\in \text{Reaction}$$ (2c)

$$0\leq v_{j}^{-}\leq -LB_{j}\;\forall j\in \text{Reaction}$$ (2d)

$$m_{k}=\sum_{j\in \text{Reaction}}\left|S_{\text{kj}}\left(v_{j}^{+}-v_{j}^{-}\right)\right|$$ (2e)

$$m_{l}=\sum_{j\in \text{Reaction}}\left|S_{\text{lj}}\left(v_{j}^{+}-v_{j}^{-}\right)\right|$$ (2f)

MCA includes the same constraints (Equations 2b–d) as FCA. Equations 2e,f, [Disp-formula e1d] are the definitions for the flux-sums of metabolite k ( $m_{k}$ ) and metabolite l ( $m_{l}$ ), respectively. The objective function maximizes or minimizes the ratio of $m_{l}$ and $m_{k}$ . The non-linear programming problem can be transformed into a mixed-integer linear programming problem (see Supplementary Material).

### Metabolite-flux coupling analysis (MetFCA)

MetFCA evaluates the ratio of the flux-sum of one metabolite and the flux through one reaction (Equation 3a).

$$\max/\min\frac{m_{l}}{v_{k}^{+}}\;or\;\left(\frac{m_{l}}{v_{k}^{-}}\right)\;\forall l\in Metabolite,\forall k\in Reaction$$ (3a)

s.t.

$$\sum_{j\in Reaction}S_{ij}\cdot \left(v_{j}^{+}-v_{j}^{-}\right)=0\;\forall i\in Metabolite$$ (3b)

$$0\leq v_{j}^{+}\leq UB_{j}\;\forall j\in \text{Reaction}$$ (3c)

$$0\leq v_{j}^{-}\leq -LB_{j}\;\forall j\in \text{Reaction}$$ (3d)

$$m_{l}=\sum_{j\in \text{Reaction}}\left|S_{\text{lj}}\left(v_{j}^{+}-v_{j}^{-}\right)\right|$$ (3e)

MetFCA includes the same constraints (Equations 3b–e) as MCA. The objective function maximizes or minimizes the ratio of $m_{l}$ and $v_{k}^{+}$ (or $m_{l}$ and $v_{k}^{-}$ ). Simplification of MetFCA formulation is shown in Supplementary Material.

### Identify blocked reactions and metabolites

Reactions that always carry zero flux are defined as “blocked” reactions. Metabolites with a constant zero flux-sum are “blocked” metabolites. To identify the blocked reactions, Flux Variability Analysis (FVA) (Mahadevan and Schilling, 2003) is performed to determine the maximal flux through each forward reaction $v_{l}^{+}$ and backward reaction $v_{l}^{-}$ (Equation 4a). The FVA formulation for each forward reaction $v_{l}^{+}$ and backward reaction $v_{l}^{-}$ is shown as follows:

$$\max v_{l}^{+}or\;\left(v_{l}^{-}\right)\;\forall l\in Reaction$$ (4a)

s.t.

$$\sum_{j\in Reaction}S_{ij}\cdot \left(v_{j}^{+}-v_{j}^{-}\right)=0\;\forall i\in Metabolite$$ (4b)

$$0\leq v_{j}^{+}\leq UB_{j}\;\forall j\in Reaction$$ (4c)

$$0\leq v_{j}^{-}\leq -LB_{j}\;\forall j\in Reaction$$ (4d)

FVA includes the same constraints (Equations 4b–d) as FCA. A forward (or backward) reaction is a blocked reaction if the maximal forward (or backward) flux is zero. A maximal flux of zero guarantees the flux is always zero because the flux through all forward and backward reactions is non-negative. Furthermore, a metabolite is considered blocked if all its associated reactions are blocked. FCA, MCA, and MetFCA are performed exclusively for each unblocked pair.

Identifying blocked reactions and metabolites is an important preprocessing step. It is meaningless to perform coupling analysis on blocked reactions or metabolites because their reaction fluxes (or metabolite flux-sums) are always zero and biologically inactive. This preprocessing step can also significantly reduce the number of pairs that need to be computed. For example, under the glucose aerobic condition using the *E. coli* iJO1366 model, there are originally 2,573 reactions (which increases to 5,146 when split into forward and backward directions). However, only 1,995 of these 5,146 forward and backward reactions remain unblocked. This means we reduce the number of pairs that need to run FCA from 13,238,085 (calculated as $\frac{5146\times 5145}{2}$ ) to 1,989,015 (calculated as $\frac{1995\times 1994}{2}$ ). This represents a reduction of more than 80% in the computational work required.

### Classification

**FIGURE 1:**
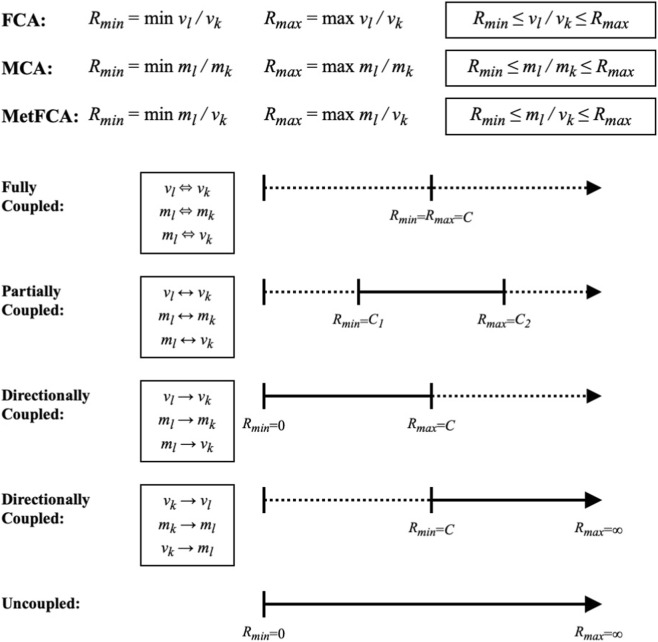
Classification of each reaction pair/metabolite pair/metabolite-reaction pair into (1) fully coupled (2) partially coupled (3) directionally coupled (4) uncoupled pair. For simplicity, $v_{l}$ and $v_{k}$ represent the flux through any arbitrary forward or backward reaction. $m_{l}$ and $m_{k}$ represent the flux-sum of any metabolite. $R_{\min}$ ( $R_{\max}$ ) is the minimum (maximum) value obtained from FCA, MCA, or MetFCA. Two elements are (1) fully coupled if a non-zero flux/flux-sum of one implies a non-zero and fixed flux/flux-sum of the other and *vice versa*, (2) partially coupled if a non-zero flux/flux-sum of one implies a non-zero flux/flux-sum of the other and *vice versa*, (3) directionally coupled if a non-zero flux/flux-sum of one implies a non-zero flux/flux-sum of the other but not *vice versa*, or (4) uncoupled if a non-zero flux/flux-sum of one implies nothing about the flux/flux-sum of the other and *vice versa*. This figure is adapted from Figure 1 in Burgard et al. (2004).

Each reaction pair, metabolite pair, metabolite-reaction pair can be classified into the following four categories: (1) fully coupled, (2) partially coupled, (3) directionally coupled, or (4) uncoupled. The detailed definition is shown in Table 2. After running FCA, MCA, and MetFCA for each unblocked pair, a minimum ratio $R_{\min}$ and a maximum ratio $R_{\max}$ are calculated. By comparing $R_{\min}$ and $R_{\max}$ , each unblocked pair can be classified into the above four categories (see Figure 1).

**TABLE 2:** Definitions of fully coupled, partially coupled, directionally coupled, uncoupled reaction pair/metabolite pair/metabolite-reaction pair.

Category	FCA for each reaction pair	MCA for each metabolite pair	MetFCA for each metabolite-reaction pair
Fully coupled	$v_{l}⟺v_{k}$ if a non-zero flux $v_{l}$ implies not only a non-zero but also a fixed flux $v_{k}$ and *vice versa*	$m_{l}⟺m_{k}$ if a non-zero flux-sum $m_{l}$ implies not only a non-zero but also a fixed flux-sum $m_{k}$ and *vice versa*	$m_{l}⟺v_{k}$ if a non-zero flux-sum $m_{l}$ implies not only a non-zero but also a fixed flux $v_{k}$ and *vice versa*
Partially coupled	$v_{l}⟷v_{k}$ if a non-zero flux $v_{l}$ implies a non-zero flux $v_{k}$ and *vice versa*	$m_{l}⟷m_{k}$ if a non-zero flux-sum $m_{l}$ implies a non-zero flux-sum $m_{k}$ and *vice versa*	$m_{l}⟷v_{k}$ if a non-zero flux-sum $m_{l}$ implies a non-zero flux $v_{k}$ and *vice versa*
Directionally coupled	$v_{l}⟶v_{k}$ if a non-zero flux $v_{l}$ implies a non-zero flux $v_{k}$ but not necessarily the reverse	$m_{l}⟶m_{k}$ if a non-zero flux-sum $m_{l}$ implies a non-zero flux-sum $m_{k}$ but not necessarily the reverse	$m_{l}⟶v_{k}$ if a non-zero flux-sum $m_{l}$ implies a non-zero flux $v_{k}$ but not necessarily the reverse
			$v_{k}⟶m_{l}$ if a non-zero flux $v_{k}$ implies a non-zero flux-sum $m_{l}$ but not necessarily the reverse
Uncoupled	Pairs not falling into one of the above categories are classified as uncoupled. A non-zero flux or flux-sum for one element implies nothing about the flux or flux-sum of the other and *vice versa*		

### Generate reaction modules, metabolite modules, and metabolite-reaction modules

Reaction modules, metabolite modules, and metabolite-reaction modules can be generated from fully coupled pairs. (1) Reaction modules: If each reaction is treated as a node, an edge can be drawn between fully coupled reaction pairs; therefore, reactions can be grouped into modules (i.e., connected components). For example, three fully coupled reaction pairs R1-R2, R2-R3, R1-R3 form one reaction module {R1, R2, R3}. Reaction modules can be converted to gene modules via the GPR associations. For example, assume the GPR of reaction R1 is “(g1 AND g2) OR g3” and the GPR of reaction R2 is g4 (where g1, g2, g3, and g4 are all genes). “g1 AND g2” indicates g1 and g2 are transcribed and translated as two separate subunits of an enzyme that together catalyze R1. The GPR for R1 indicates that this enzyme complex and the enzyme formed from g3 are isoenzymes that can independently catalyze R1. If reaction R1 and R2 form a reaction module {R1, R2}, then the resulting gene module is {g1, g2, g3, g4}. (2) Metabolite modules: If each metabolite is viewed as a node, an edge can be drawn between fully coupled metabolite pairs. Therefore, metabolites can be grouped into modules. (3) Metabolite-reaction modules: If each metabolite and each reaction are viewed as a node, there are three kinds of edges in this graph: (1) edges between fully coupled metabolite and reaction pairs, (2) edges between fully coupled reaction pairs, and (3) edges between fully coupled metabolite pairs. These metabolite-reaction modules can be converted to metabolite-gene modules via GPR associations, as described above. A comparison between the above three modules is shown in Table 3.

**TABLE 3:** Comparison between reaction modules, metabolite modules, and metabolite-reaction modules.

Property	Reaction modules	Metabolite modules	Metabolite-reaction modules
Nodes	Reactions	Metabolites	Reactions and metabolites
Edges	Between fully coupled reaction pairs	Between fully coupled metabolite pairs	(1) Between fully coupled metabolite and reaction pairs (2) between fully coupled reaction pairs (3) between fully coupled metabolite pairs
Generated by	FCA	MCA	MetFCA
Converted to gene modules	Yes if GPR associations are available	No	Yes if GPR associations are available
Used to analyze	Transcriptomics and/or proteomics data	Metabolomics data	Transcriptomics and/or proteomics and metabolomics data

We used the MATLAB package “networkComponents” 1.2.0.0 (Larremore, 2026) to find the modules from the FCA, MCA, and MetFCA fully coupled pairs. This package implements a search algorithm to identify modules. For example, if we have these fully coupled metabolite pairs after running MCA, A-B, B-C, A-C, E-F, G-H, the algorithm returns 3 metabolite modules ({A,B,C}, {E,F}, {G,H}).

### Procedure

We developed a procedure to generate all reaction modules, metabolite modules, and metabolite-reaction modules (gene modules and metabolite-gene modules if GPR associations are available) for any GEM.

Step 1: Find all unblocked reactions (forward or backward reactions) and metabolites under a specific medium condition for a wildtype or mutant strain (Equations 4a–d).
Step 2:
  2a. Run FCA (Equations 1a–d) for every unblocked reaction pair using the same $UB_{j}$ and ${LB}_{j}$ as Step 1. $R_{\min}$ and $R_{\max}$ are obtained.
  2b. Run MCA (Equations 2a–f) for every unblocked metabolite pair using the same $UB_{j}$ and ${LB}_{j}$ as Step 1. $R_{\min}$ and $R_{\max}$ are obtained.
  2c. Run MetFCA (Equations 3a–e) for every unblocked metabolite-unblocked reaction pair using the same $UB_{j}$ and ${LB}_{j}$ as Step 1. $R_{\min}$ and $R_{\max}$ are obtained.
Step 3: Compare $R_{\min}$ and $R_{\max}$ , and classify each pair as fully coupled, partially coupled, directionally coupled, or uncoupled (see Figure 1).
Step 4: Generate reaction modules, metabolite modules, and metabolite-reaction modules for fully coupled pairs.
Step 5: (Optional): Convert reaction modules or metabolite-reaction modules to gene modules or metabolite-gene modules, respectively, if GPR associations are available.

### Data acquisition

Three GEMs were utilized in this study: a custom-built toy network developed specifically for this analysis, the iJO1366 (Orth et al., 2011) GEM and the *iHsa* (Blais et al., 2017) GEM which were obtained from their Supplementary Material. The omics data used consisted of a transcriptomics and metabolomics dataset measured in human Calu-3 cells infected with the influenza virus (H7N9). This dataset was provided by collaborators and is publicly available for download at the PNNL Datahub (identifier: ICL102) (Eisfeld et al., 2024).

All computational analyses were performed using specialized software tools. FCA, MCA, and MetFCA were solved using the CPLEX solver in GAMS, while classification and module generation were executed in MATLAB R2018a.

### Omics data analysis using modules generated by coupling analysis

To analyze the transcriptomics dataset mentioned above, we utilized the gene modules derived from the human GEM *iHsa*. The dataset comprised measurements from mock-infected and influenza-infected Calu-3 cell lines at four time points (0, 7, 12, and 24 h post-infection). For each gene at each time point, the Log_2_ transcriptomic fold change between the infected and mock-infected cells $Log_{2}\left(\frac{influenza-infected\;}{mock-infected}\right)$ was calculated. Finally, Gene Set Enrichment Analysis (GSEA) (Subramanian et al., 2005) was performed using the Python package GSEApy v0.9.3 (Fang et al., 2023). Statistical significance for enriched modules was defined as a False Discovery Rate (FDR) < 0.05 to correct for multiple hypothesis testing and strictly limit the expected proportion of false-positive findings.

### Comparison of modules generated by WGCNA and coupling analysis

Data-driven approaches such as WGCNA (Langfelder and Horvath, 2008), oCEM (Nguyen and Le, 2022), MEGENA (Song and Zhang, 2015), and CEMiTool (Russo et al., 2018; Cardozo et al., 2019) can generate gene modules relying solely on transcriptomic data. For comparison, we applied WGCNA to the previously described transcriptomics dataset using the Python package pyWGCNA v2.1.3 (Rezaie et al., 2023) with default parameters. We subsequently mapped every gene within the WGCNA gene modules and the FCA gene modules to KEGG pathways, noting that a single gene can map to multiple pathways.

## Results

### FCA, MCA, and MetFCA results for the toy network

**FIGURE 2:**
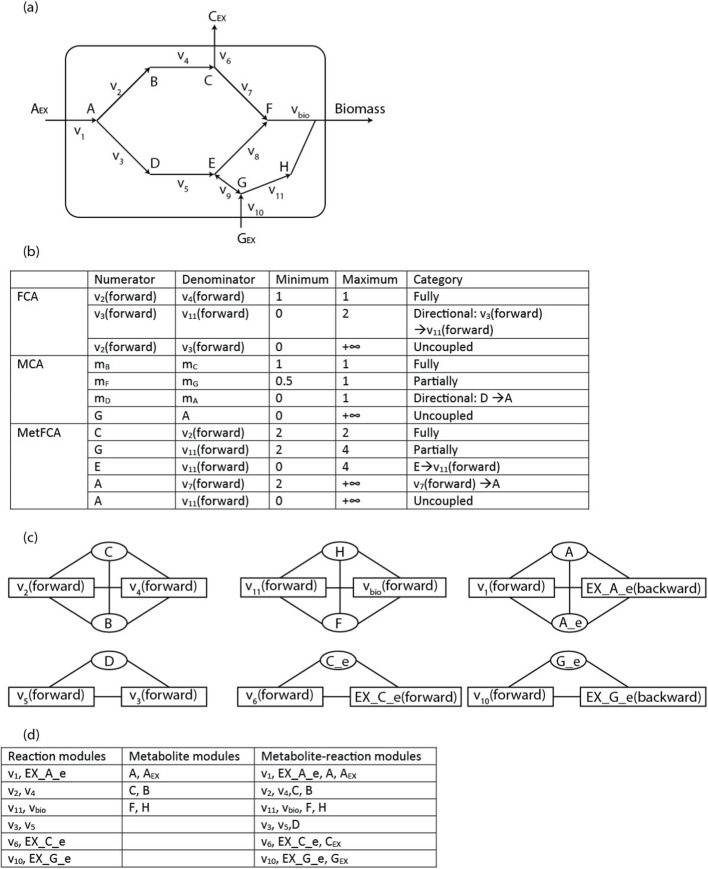
Results for the toy network. **(a)** Toy network with 11 metabolites and 15 reactions. (Reproduced from Figure 2a in Tervo and Reed (2012)) **(b)** Examples of fully/partially/directionally coupled pairs from FCA, MCA, and MetFCA. **(c)** Graphs of metabolite-reaction modules for this toy network. Metabolite nodes are represented as ellipses. Reaction nodes are represented as rectangles. **(d)** Reaction modules, metabolite modules, and metabolite-reaction modules for this toy network.

We performed FCA, MCA, and MetFCA on the toy network (see Figure 2a). This network has 11 metabolites and 15 reactions (or 30 reactions if forward and backward reactions are considered as two separate reactions). These 15 reactions are $v_{1}∼\;v_{11},v_{bio},\text{EX}\_\text{A}\_\text{e},\text{EX}\_\text{G}\_\text{e},\text{and EX}\_\text{C}\_\text{e}$ . Among these 30 reactions (including both the forward and backward directions), 16 of them are unblocked reactions (forward: $v_{1}∼\;v_{11},v_{bio},\text{EX}\_C\_e$ ; backward: $v_{9},EX\_A\_e,EX\_G\_e$ ) when A and G are only allowed to be taken up, and C is only allowed to be secreted. All 11 metabolites are unblocked. We performed FCA, MCA and MetFCA for each unblocked pair, and classified each pair based on the minimum and maximum of the ratio. Examples of the results are shown in Figure 2b. There are 6 metabolite-reaction modules for the toy network (see Figure 2c). The resulting reaction modules, metabolite modules, and metabolite-reaction modules are shown in Figure 2d.

Why are B and C fully coupled? Assume $v_{2}=1$ , due to the steady-state constraint of metabolite B, $v_{2}=v_{4}=1$ and this ensures there is no accumulation of B. Consequently, the flux-sums $m_{B}=m_{C}=2$ . Therefore, the ratio $\frac{m_{B}}{m_{C}}=1$ . A non-zero flux-sum of B implies not only a non-zero but also a fixed flux-sum of C and *vice versa*, indicating full coupling.

Why are F and G partially coupled? Assume $v_{bio}=1$ , we have $v_{7}+v_{8}=v_{bio}=1$ , $v_{11}=1$ (due to steady-state constraints of F and H), and $m_{F}=2$ . There are two extreme cases:

Extreme Case 1: If $v_{9}=0$ , then $m_{G}=2$ , making $\frac{m_{F}}{m_{G}}=1$ .

Extreme Case 2: If $v_{9}=1$ (direction from G to E), then $m_{G}=4$ , resulting in $\frac{m_{F}}{m_{G}}=0.5$ .

$\frac{m_{F}}{m_{G}}$ is between 0.5 and 1. This variability indicates partial coupling because a non-zero flux-sum of F implies a non-zero flux-sum of G and *vice versa*.

Why are D and A directionally coupled (D → A)? When there is flux through D ( $m_{D}>0$ ), this means $v_{3}\;>\;0$ and hence $m_{A}>0$ . A non-zero flux-sum of D implies a non-zero flux-sum of A but not necessarily the reverse.

Why are G and A uncoupled? G and A are both medium components. The cell can uptake them independently in this toy network. For example:

- The cell can only take up A to produce F and H for growth.
- Alternatively, it can only take up G to produce F and H for growth.
- Or it can take up both A and G to produce F and H for growth.

Since a nonzero flux-sum of A does imply nothing about the flux-sum of G, and *vice versa*, G and A are uncoupled.

### FCA, MCA, and MetFCA results for *E. coli* iJO1366

**FIGURE 3:**
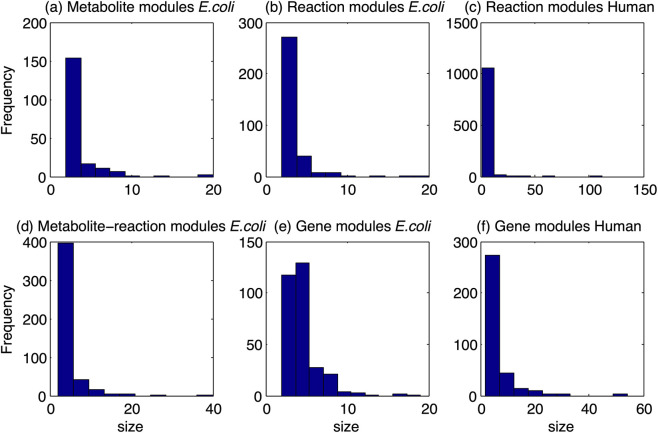
Module size distribution for reaction modules, metabolite modules, metabolite-reaction modules, and gene modules in iJO1366 and *iHsa*. Panels **(a,b,d,e)** show histograms for the numbers of metabolites, reactions, or genes in modules identified for iJO1366 under the glucose aerobic minimal medium condition. Panels **(c,f)** show histograms for the numbers of reactions or genes in modules identified for *iHsa* when all metabolites with exchange reactions were allowed to be freely taken up or secreted. Gene modules containing one gene were not included in the histograms. For *E. coli*, most of the modules have a size of less than 5. Largest module sizes of reaction modules, metabolite modules, metabolite-reaction modules, and gene modules are 20, 20, 40, and 19, respectively. For human, largest module sizes of reaction modules and gene modules are 112 and 54, respectively. The predominance of small modules in both the *E. coli* and human GEMs indicates highly flexible metabolism. Because reactions/genes/metabolites are rarely locked into large, fully coupled modules, the metabolic network can easily reroute fluxes in response to changing conditions.

We also performed FCA, MCA, and MetFCA on the *E. coli* iJO1366 model (Orth et al., 2011). The model has 1366 genes, 2573 metabolic reactions (including the exchange reactions), and 1803 metabolites. We incorporated additional exchange reactions to produce each biomass component independently, because the biomass reaction is artificial and creates unnecessarily large modules. We set the upper and lower bounds accordingly for each exchange reaction to simulate a glucose aerobic condition. Under this condition there are 1995 unblocked reactions (after splitting each reaction to forward and backward directions) and 1155 unblocked metabolites. The detailed results for FCA, MCA, and MetFCA are shown in Table 4. For all three coupling methods, most of the pairs (≥96%) are uncoupled. There are 330 reaction modules (containing 899 forward and backward reactions, 17.5% of all 5146 forward and backward reactions in iJO1366), 192 metabolite modules (containing 602 metabolites, 33.4% of all 1803 metabolites in iJO1366), and 464 metabolite-reaction modules. We converted these 330 reaction modules to 305 gene modules, which contain 499 genes (36.5% of all 1366 genes in iJO1366). We could not convert some reaction modules to gene modules because (1) some reactions do not have genes associated with them according to GPR associations, and (2) we excluded gene modules with only one gene. The size distributions of these modules are shown in Figure 3. These modules are provided in Supplementary Table 1.

**TABLE 4:** Results of FCA, MCA, and MetFCA on *E. coli* iJO1366 model.

Type of pairs	FCA	MCA	MetFCA
Fully coupled pairs	1,555	1,237	3,171
Partially coupled pairs	105	114	207
Directionally coupled pairs	17,459	24,789	9,278 ( m⟶v )^a^
			39,158 ( v⟶m )^b^
Uncoupled pairs	1,969,896 (99%)^c^	640,295 (96%)^c^	2,252,411 (98%)^c^
Total pairs	1,989,015	666,435	2,304,225

Total pairs are all the unblocked pairs that FCA, MCA, and MetFCA, looped through.

^a^ 9,278 pairs are directionally coupled ( metabolite⟶reaction ).

^b^ 39,158 pairs are directionally coupled ( reaction⟶metabolite ).

^c^ Percentage of uncoupled pairs among total pairs.

**FIGURE 4:**
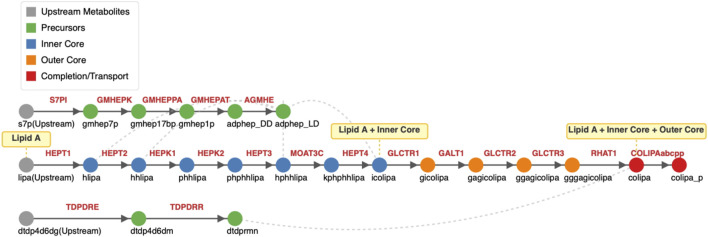
Modules for lipopolysaccharide biosynthesis pathway in *E. coli* iJO1366 model. Reactions, indicated as capital letters, are fully coupled and constitute a distinct reaction module. Similarly, circled metabolites (excluding those designated as upstream metabolites) are fully coupled, forming a metabolite module. Together, these elements comprise a comprehensive metabolite-reaction module that maps directly to the lipopolysaccharide biosynthesis pathway.

The largest metabolite-reaction module (Figure 4) comprises a 20-reaction, 20-metabolite non-stop segment of the lipopolysaccharide (LPS) biosynthesis pathway (KEGG map00540) (Kanehisa, 2000). In *E. coli*, this pathway dictates the assembly of colipa, the complete core oligosaccharide-lipid A molecule. The process initiates by synthesizing specialized nucleotide sugar precursors (adphep_LD) to build the inner core onto the KDO2-lipid A (lipa), forming the intermediate icolipa. Common hexoses (glucose, galactose, and rhamnose) are then sequentially added to form the outer core, completing the colipa macromolecule for transport. All reaction and metabolite identifiers match those in the BiGG Models database (King et al., 2016). Ultimately, the isolation of this functionally cohesive module highlights the capacity of FCA, MCA and MetFCA to successfully capture biologically meaningful pathways without any unrelated reactions and metabolites.

The FSCA method (Seyis et al., 2025) shares the same theoretical formulation as MCA but employs a different computational approach. In the FSCA paper, the authors compared FSCA with MCA for iJO1366. FSCA identified 257 fully coupled pairs, 2,311 partially coupled pairs, and 555,831 directionally coupled pairs. In contrast, MCA found 1,237 fully coupled pairs, 114 partially coupled pairs, and 24,789 directionally coupled pairs. This discrepancy likely arises because the MCA results in Table 4 were generated under the glucose aerobic condition, whereas FSCA might have been evaluated using a different, unspecified culture medium. Furthermore, the fully coupled metabolite pairs identified for *E. coli* iML1515 (Monk et al., 2017) using FSCA show some overlap with those found using MCA on *E. coli* iJO1366. While differences between the two GEMs result in some variations among the exact members of each module, both methods successfully identify similar functional modules. Key shared findings include:

- LPS biosynthesis pathway: Both methods identify this pathway as containing the highest number of fully coupled pairs. Notably, MCA groups these into a single large metabolite module (Figure 4), while FSCA resolves them into two smaller modules.
- Tyrosine, tryptophan, and phenylalanine metabolism: Both approaches identify an identical four-metabolite module (2cpr5p, 3ig3p, anth, pran).
- Cell envelope biosynthesis: Both methods successfully capture this pathway (dtdp4aaddg, dtdp4addg, eca2und_p, eca3und_p, eca4colipa_e, eca4colipa_p, eca4und_p, uacmam, uacmamu, udcpdp_p, unaga, unagamu, unagamuf, unagamuf_p). As with the LPS pathway, MCA identifies one comprehensive module, whereas FSCA divides the pathway into two smaller modules.

### FCA, MCA, and MetFCA results for human *iHsa*

We performed FCA on the human metabolic model *iHsa* (Blais et al., 2017). *iHsa* contains 5620 metabolites, 8263 reactions, and 2315 genes, and describes metabolic capabilities across all human cell types. All metabolites with exchange reactions were allowed to be freely taken up or secreted. We treated the biomass components in the same way as in the *E. coli* iJO1366 model. There are 8082 unblocked reactions (including forward and backward reactions) and 4140 unblocked metabolites. After running FCA, 1078 reaction modules were generated. These 1078 reaction modules were converted to 353 fully coupled gene modules, which contain 1040 genes (45% of 2315 genes in *iHsa*). On average, a gene module contains 6.3 genes, with the largest gene module containing 54 genes. The size distributions of the reaction and gene modules are shown in Figure 3.

Due to computational constraints—performing MCA for a single metabolite against all unblocked metabolites took approximately 6 hours—we restricted our MCA and MetFCA to a subset of 274 common human metabolites. According to the Human Metabolome Database (HMDB) (Wishart et al., 2018), there are 582 endogenous cytoplasm metabolites that can be detected and quantified. Only 274 of these 582 metabolites are within the *iHsa* model. We performed MCA on these 274 common metabolites with all 4140 unblocked metabolites and found 67 metabolite modules. We also ran MetFCA on these common metabolites with all unblocked reactions and identified 84 metabolite-reaction modules. These modules are provided in Supplementary Table 2.

### Omics data analysis using gene modules derived from *iHsa*

We used the 353 gene modules derived from *iHsa* to analyze the previously described transcriptomics dataset with GSEA. Three gene modules with at least 10 genes were found to be significantly enriched and downregulated in influenza-infected cells compared to mock-infected cells at 24 h. These three gene modules are involved in beta oxidation of fatty acids (FDR = 0.011), proteoglycan metabolism (FDR = 0.0482), and omega-6 fatty acid metabolism (FDR = 0.0448). Alongside the transcriptomics data, we analyzed a co-measured metabolomics dataset. This dataset is limited to 65 metabolites, with only 47 mapping to the *iHsa* model. Of those 47, very few appear in the metabolite or metabolite-gene modules, making the data too sparse for enrichment analysis. While standard GSEA guidelines recommend a minimum module size of 15 elements, we relaxed this threshold to 10 for the fully coupled metabolite modules. We reasoned that within these tightly linked networks, significant changes in just two or three metabolites are sufficient to indicate functional enrichment. However, even with this relaxed criterion, the dataset lacks a single metabolite module containing 10 or more measured metabolites.

### Comparison of modules generated by WGCNA and coupling analysis

**FIGURE 5:**
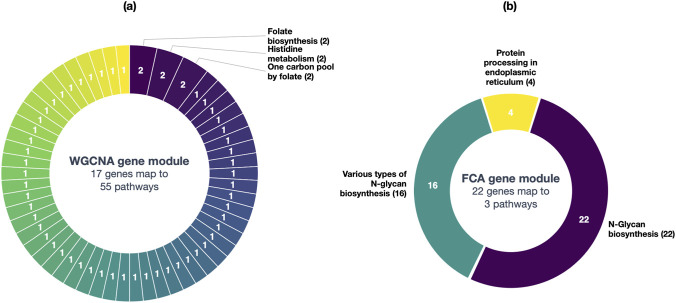
Pathway distribution of gene modules generated by WGCNA and FCA. **(a)** Pathway distribution of the 17-gene module generated by WGCNA. **(b)** Pathway distribution of the 22-gene module generated by FCA. The FCA gene module has a clear functional meaning, while the WGCNA gene module contains scattered genes from unrelated pathways. The WGCNA module contains 17 genes (138050, 79868, 9120, 8382, 1551, 26958, 5728, 1719, 144193, 1719, 54898, 445, 23363, 4128, 9653, 5825, 1787), and the FCA module contains 22 genes (10195, 144245, 1603, 1650, 1798, 199857, 29929, 4122, 4124, 4245, 4247, 440138, 56052, 57171, 6184, 6185, 79053, 79087, 79796, 79868, 84920, 85365). Gene IDs are provided as Entrez Gene IDs. Every gene was mapped to one or more pathways when available in KEGG, and occurrences of each unique pathway name were counted. The WGCNA module mapped to 55 pathways, among which three pathways occurred two times and 52 pathways occurred once (pathway names are not shown in the figure). In contrast, the 22-gene FCA module mapped to *N-Glycan biosynthesis* 22 times, *Various types of N-glycan biosynthesis* 16 times, and *Protein processing in endoplasmic reticulum* 4 times, reflecting precisely that it is involved in N-glycan biosynthesis.

Applying WGCNA to the previously described transcriptomics dataset yielded nine gene modules in total: eight containing 90 or more genes, and one containing 17 genes. Using the 17-gene module as a representative example (Figure 5a), the associated KEGG pathways were highly diverse and scattered across unrelated functions. Although we highlight only one of the nine WGCNA modules, the remaining modules exhibited a similar trend; because they lack a dominant, unifying function or pathway, the biological significance of these modules remains unclear.

In contrast, modules generated by FCA demonstrated high functional concentration and clarity. For comparison, we selected an FCA module of comparable size (22 genes). As shown in Figure 5b, the genes within this module predominantly mapped to three specific KEGG pathways: *N-Glycan biosynthesis*, *Various types of N-glycan biosynthesis*, and *Protein processing in endoplasmic reticulum*, strongly indicating a precise role in N-glycan biosynthesis. We restricted this comparative analysis to FCA gene modules due to the previously mentioned dataset limitations—specifically, an insufficient number of shared metabolites between the metabolomics data and the human *iHsa*.

## Conclusion

In this paper, we described two methods, MCA and MetFCA, which evaluate the dependencies between metabolites, as well as between metabolites and reactions. Along with FCA, these two coupling methods can generate various modules under any medium conditions or genetic perturbations. Both methods are based on mixed-integer programming after transforming the non-linear problem into a linear problem, which can be solved in a reasonable time for the GEMs similar size to *E. coli*. Solving the human GEM is challenging because the number of pairs ( $\frac{n\left(n-1\right)}{2}$ ) increases quadratically with metabolite size *n* and solving each pair also takes longer because the human GEM is more complex.

MCA and MetFCA assume cells are operating at steady-state, which means metabolite concentrations are time-invariant. This is a valid assumption, even for virus-infected cells because cell growth rate and virus replication rate are much slower than metabolism dynamics (Tian et al., 2017). This assumption simplifies the model formulation because researchers do not have to deal with the dynamics properties of metabolic networks, which requires kinetic parameters and large systems of ordinary differential equations.

We applied FCA, MCA, and MetFCA to three GEMs: the toy network, the *E. coli* iJO1366, and human *iHsa*. The resulting modules are shown in Supplementary Tables 1, 2. For iJO1366, fully coupled, partially coupled, and directionally coupled pairs account for a very small portion of the total unblocked pairs (<5%). Fully coupled pairs can form modules, which can be used to analyze omics datasets. We showed one case study involving human metabolism where gene modules were used to analyze transcriptomics data. Although the limited metabolite coverage in this dataset precludes a similar demonstration here, metabolite modules can be applied to metabolomics data analysis. Furthermore, the metabolite-gene modules established here provide a framework for researchers to jointly analyze transcriptomic, proteomic, and metabolomic data. The identified modules are dependent on experimental conditions, since fluxes through reactions depend on what substrates are available for cells to consume. In our human case study, it is unknown which metabolites were exchanged between the rich medium and the cells, so all metabolites with exchange reactions were allowed to be freely taken up or secreted. Consequently, given the same human GEM, the fully coupled modules identified here should remain coupled across all possible medium conditions. However, if medium components are experimentally measured, condition-specific modules can be generated to provide more targeted biological insights.

For this study, module generation is restricted to fully coupled pairs. As demonstrated in Figures 4, 5, these pairs provide the most robust evidence that the resulting modules operate within a specific metabolic pathway and correspond to distinct biological functions. In a fully coupled pair, an increase in one flux/flux-sum guarantees a corresponding increase in the other. Therefore, the omics data are expected to change in the same direction (i.e., both overexpressed or both underexpressed). Partially and directionally coupled pairs are excluded from the omics data analysis, because their flux/flux-sum changes may not be in the same direction. For example, if reaction 1 and reaction 2 are partially coupled with $1\leq \frac{{\;v}_{1}}{v_{2}}\leq 2$ , the two states $v_{1}=v_{2}=1.5$ and $v_{1}=2,v_{2}=1$ both satisfy the ratio. However, shifting between them results in $v_{1}$ increasing and $v_{2}$ decreasing. Furthermore, uncoupled pairs are also omitted, as a non-zero flux or flux-sum in one element does not imply any relevance to another.

## Data Availability

The code for MCA and MetFCA is available at https://github.com/mtian29/metabolite_coupling_methods.
