# Supplementary material for: Metabolite coupling analysis and metabolite-flux coupling analysis of genome-scale metabolic models
Source: Front Bioinform. 2026 Jul 1;6:1859473. doi: 10.3389/fbinf.2026.1859473 (PMC13370160; doi:10.3389/fbinf.2026.1859473)
Supplement: Supplementary file 2 [file DataSheet1.pdf]

Supplemental Information

1. Simplification of the FCA formulation

The Flux Coupling Analysis (FCA) formulation was shown in **Equations (1a–1d)** of the paper. $\mathrm{Max/min}\ \frac{\mathrm{v_l^+}}{\mathrm{v_k^+}}$ is used as an example to show how to simplify the formulation.

$\mathrm{Max}\ \frac{\mathrm{v_l^+}}{\mathrm{v_k^+}}$ is equivalent to $\min \frac{\mathrm{v_k^+}}{\mathrm{v_l^+}}$. Consequently, the objective function becomes $\min \frac{\mathrm{v_k^+}}{\mathrm{v_l^+}}$ and $\min \frac{\mathrm{v_l^+}}{\mathrm{v_k^+}}$. The denominator is further set to 1 to remove it from the objective function.

To obtain $R_{max} = \max \frac{\mathrm{v_l^+}}{\mathrm{v_k^+}} = \frac{1}{\min \frac{\mathrm{v_k^+}}{\mathrm{v_l^+}}}$, the following optimization problem was solved for every reaction pair (k, l) :

$\min\ v_k^+ \quad \forall k, l \in Reaction$ (S1a)

s.t.

$\sum_{j \in Reaction} S_{ij} \cdot (v_j^+ - v_j^-) = 0 \qquad \forall i \in Metabolite$ (S1b)

$0 \leq v_j^+ \leq UB_j \quad \forall j \in Reaction$ (S1c)

$0 \leq v_j^- \leq -LB_j \quad \forall j \in Reaction$ (S1d)

$v_l^+ = 1$ (S1e)

After solving **Equations (S1a–S1e)**, R_{max} was calculated as $\frac{1}{objective\ function\ from\ S1a}$

($R_{max} = +\infty$, if the objective function from **Equation S1a** is 0).

To obtain $R_{min} = \min \frac{\mathrm{v}_\mathrm{l}^+}{\mathrm{v}_\mathrm{k}^+}$, the following optimization problem was solved for every reaction pair (k, l) as before:

$$\min\ v_l^+ \quad \forall k, l \in Reaction \tag{S2a}$$

s.t.

$$\sum_{j \in Reaction} S_{ij} \cdot (v_j^+ - v_j^-) = 0 \qquad \forall i \in Metabolite \tag{S2b}$$

$$0 \le v_j^+ \le UB_j \ \ \forall j \in Reaction \tag{S2c}$$

$$0 \le v_j^- \le -LB_j \quad \forall j \in Reaction \tag{S2d}$$

$$v_k^+ = 1 \tag{S2e}$$

After solving **Equations (S2a–S2e)**, R_{min} was calculated as $objective\ function\ from\ S2a$. These formulations (**Equations S1a–S1e, S2a–S2e**) became linear programming problems.

2. Simplification of the MCA formulation

The Metabolite Coupling Analysis (MCA) formulation was shown in **Equations (2a–2f)** of the paper. $\mathrm{Max}\ \frac{\mathrm{m_l}}{\mathrm{m_k}}$ is equivalent to $\min\ \frac{m_k}{\mathrm{m_l}}$. Consequently, the objective function becomes $\min\ \frac{m_k}{\mathrm{m_l}}$ and $\min \frac{\mathrm{m_l}}{\mathrm{m_k}}$. The denominator is further set to 1 to remove it from the

objective function. To remove the absolute value terms in m_k and m_l, a binary variable z_j is introduced to ensure that the flux through at least one of the forward reaction v_j^+ and the backward reaction v_j^- is zero.

To obtain $R_{max} = \max \frac{\mathrm{m_l}}{\mathrm{m_k}} = \frac{1}{\min \frac{m_k}{\mathrm{m_l}}}$, the following optimization problem was solved for every metabolite pair (k, l) :

$\min m_k \;\; \forall l, k \in Metabolite$ (S3a)

s.t.

$\sum_{j \in Reaction} S_{ij} \cdot (v_j^+ - v_j^-) = 0 \qquad \forall i \in Metabolite$ (S3b)

$0 \le v_j^+ \le z_j \cdot UB_j \;\; \forall j \in Reaction$ (S3c)

$0 \le v_j^- \le -(1 - z_j) \cdot LB_j \quad \forall j \in Reaction$ (S3d)

$m_l = \sum_{j \in Reaction} |S_{lj}| (v_j^+ + v_j^-) = 1$ (S3e)

$z_j \in \{0,1\} \quad \forall j \in Reaction$ (S3f)

After solving **Equations (S3a–S3f)**, R_{max} was calculated as $\frac{1}{objective\ function\ from\ S3a}$ ($R_{max} = +\infty$,if the objective function from **Equation S3a** is 0).

To obtain $R_{min} = \min \frac{\mathrm{m_l}}{\mathrm{m_k}}$, the following optimization problem was solved for every metabolite pair (k, l) as before:

$\min m_l \ \ \forall l, k \in Metabolite$ (S4a)

s.t.

$\sum_{j \in Reaction} S_{ij} \cdot (v_j^+ - v_j^-) = 0 \qquad \forall i \in Metabolite$ (S4b)

$0 \leq v_j^+ \leq z_j \cdot UB_j \ \ \forall j \in Reaction$ (S4c)

$0 \leq v_j^- \leq -(1 - z_j) \cdot LB_j \quad \forall j \in Reaction$ (S4d)

$m_k = \sum_{j \in Reaction} |S_{kj}| (v_j^+ + v_j^-) = 1$ (S4e)

$z_j \in \{0,1\} \quad \forall j \in Reaction$ (S4f)

After solving **Equations (S4a–S4f)**, R_{min} was calculated as $objective\ function\ from\ S4a$. These formulations (**Equations S3a–S3f, S4a–S4f**) became mixed-integer linear programming problems.

3. Simplification of the MetFCA formulation

The Metabolite-flux Coupling Analysis (MetFCA) formulation was shown in **Equations (3a–3e)** of the paper. $Max/min \frac{m_l}{v_k^+}$ is used as an example to show how to simplify the formulation. $\text{Max} \frac{m_l}{v_k^+}$ is equivalent to $\min \frac{v_k^+}{m_l}$. Consequently, the objective function becomes $\min \frac{v_k^+}{m_l}$ and $\min \frac{m_l}{v_k^+}$. The denominator is further set to 1 to remove it from the objective function. To remove the absolute value term in m_k, a binary variable z_j is

introduced to ensure that the flux through at least one of the forward reaction v_j^+ and the backward reaction v_j^- is zero.

To obtain $R_{max} = \max \frac{m_l}{v_k^+} = \frac{1}{\min \frac{v_k^+}{m_l}}$, the following optimization problem was solved for every metabolite-reaction pair (l, k) :

$$\min v_k^+ \;\; \forall l \in Metabolite, \forall k \in Reaction \tag{S5a}$$

s.t.

$$\sum_{j \in Reaction} S_{ij} \cdot (v_j^+ - v_j^-) = 0 \qquad \forall i \in Metabolite \tag{S5b}$$

$$0 \leq v_j^+ \leq z_j \cdot UB_j \;\; \forall j \in Reaction \tag{S5c}$$

$$0 \leq v_j^- \leq -(1 - z_j) \cdot LB_j \quad \forall j \in Reaction \tag{S5d}$$

$$m_l = \sum_{j \in Reaction} \left| S_{lj} \right| (v_j^+ + v_j^-) = 1 \tag{S5e}$$

$$z_j \in \{0,1\} \quad \forall j \in Reaction \tag{S5f}$$

After solving **Equations (S5a–S5f)**, R_{max} was calculated as $\frac{1}{objective\ function\ from\ S5a}$ ($R_{max} = +\infty$, if the objective function from **Equation S5a** is 0).

To obtain $R_{min} = \min \frac{m_l}{v_k^+}$, the following optimization problem was solved for every metabolite-reaction pair (l, k) :

$$\min m_l \;\; \forall l \in Metabolite, \forall k \in Reaction \tag{S6a}$$

s.t.

$$\sum_{j \in Reaction} S_{ij} \cdot (v_j^+ - v_j^-) = 0 \quad \forall i \in Metabolite \tag{S6b}$$

$$0 \le v_j^+ \le z_j \cdot UB_j \ \ \forall j \in Reaction \tag{S6c}$$

$$0 \le v_j^- \le -(1 - z_j) \cdot LB_j \quad \forall j \in Reaction \tag{S6d}$$

$$m_l = \sum_{j \in Reaction} |S_{lj}| (v_j^+ + v_j^-) \tag{S6e}$$

$$z_j \in \{0,1\} \quad \forall j \in Reaction \tag{S6f}$$

$$v_k^+ = 1 \tag{S6g}$$

After solving **Equations (S6a–S6g)**, R_{min} was calculated as $objective\ function\ from\ S6a$. These formulations (**Equations S5a–S5f, S6a–S6g**) became mixed-integer linear programming problems.

There is a simpler formulation to obtain $R_{min} = \min \frac{m_l}{v_k^+}$. In this formulation, we use the absolute value trick to avoid introducing the binary variable z_j.

$$\min m_l \ \ \forall l \in Metabolite, \forall k \in Reaction \tag{S7a}$$

s.t.

$$\sum_{j \in Reaction} S_{ij} \cdot (v_j^+ - v_j^-) = 0 \quad \forall i \in Metabolite \tag{S7b}$$

$$0 \le v_j^+ \le UB_j \ \ \forall j \in Reaction \tag{S7c}$$

$$0 \le v_j^- \le -LB_j \quad \forall j \in Reaction \tag{S7d}$$

$$m_l = \sum_{j \in Reaction} \left| S_{lj} \right| (v_j^+ + v_j^-) \tag{S7e}$$

$$v_k^+ = 1 \tag{S7f}$$

The formulation (**S7a–S7f**) ensures that at least one of the v_j^+ and v_j^- is zero because $\sum_{j \in Reaction} \left| S_{lj} \right| (v_j^+ + v_j^-)$ is minimized. If v_j^+ and v_j^- are both nonzero, the minimum of v_j^+ and v_j^- can be subtracted from both v_j^+ and v_j^- ; therefore, the value of the objective function becomes smaller while v_j^+ and v_j^- remain feasible. The formulation (**S7a–S7f**) to obtain $R_{min} = \min \frac{m_l}{v_k^+}$ is a linear programming problem.
